# High horizontal force and lateral force transmission induced by the twist of the Achilles tendon

**DOI:** 10.3389/fbioe.2026.1874501

**Published:** 2026-07-23

**Authors:** Shota Enomoto, Kohta Ito

**Affiliations:** 1 Graduate School of Education, Okayama University, Okayama, Japan; 2 Artificial Intelligence Research Center, National Institute of Advanced Industrial Science and Technology (AIST), Tokyo, Japan

**Keywords:** computer aided engineering, finite element analysis, modeling, morphology, subtendons

## Abstract

The Achilles tendon—the largest and strongest of the many tendons in the human body—consists of subtendons arising from each head of the triceps surae. These subtendons run helically and intertwine to form a twisted three-dimensional structure. More than a century has passed since the twisted structure of the Achilles tendon was first described, and numerous studies have investigated its functional morphology. However, it remains unclear how the presence or degree of twist affects the mechanical function of the lower limbs. Here, using finite element models of the Achilles tendon with and without twist, we show that the presence and degree of twist can modulate the forces transmitted from muscle to skeleton in a complex manner. In contrast to the model without twist, the twisted models clearly exhibit anterolaterally directed horizontal forces at the distal end under a simple proximal tensile load. The lateral and anterior components reach approximately 101 N and 63 N, respectively. Furthermore, the twisted structure induces inter-subtendon force transmission, and the magnitude of the transmitted force increases with increasing degree of twist. These results indicate that the uniaxial contractile force generated by the triceps surae is transformed into triaxial forces by the twisted Achilles tendon and is redistributed in a complex three-dimensional manner through inter-subtendon interactions.

## 1 Introduction

Tendons are vital organs that effectively transmit contractile muscular forces to the skeleton and generate joint motion. In particular, the Achilles tendon—the largest and strongest in the human body—contributes to the generation of ankle joint torque and associated propulsive forces, thereby playing a crucial role in fundamental human movements such as walking and running. Therefore, substantial efforts have been devoted to elucidating the function and morphology of the Achilles tendon (Finni and Vanwanseele, 2023). However, the unique twisted structure of the Achilles tendon, which is not observed in other tendons, complicates the mechanical interpretation of its function (Łazarz et al., 2024).

The Achilles tendon consists of subtendons arising from the three components of the triceps surae: the lateral (LG) and medial (MG) heads of the gastrocnemius, and the soleus (SOL). These subtendons run helically and intertwine to form a twisted three-dimensional structure (Edama et al., 2015). More than a century has passed since the twisted structure of the Achilles tendon was first described (Parsons, 1894). Cadaveric studies have consistently reported that (i) Achilles tendon twist is observed in all individuals; (ii) the direction of twist is conserved across individuals, being counterclockwise (clockwise) when the right (left) Achilles tendon is viewed from its proximal end to its distal end; and (iii) the degree of twist exhibits substantial variability among individuals (Edama et al., 2015; Pękala et al., 2017; Prosenz et al., 2018; Szaro et al., 2009; van Gils et al., 1996). As the generality of the twisted structure of the human Achilles tendon has become evident, research interest has shifted toward elucidating the necessity of this morphology and its functional significance.

In recent years, increased effort has been made to directly elucidate why the Achilles tendon exhibits such a complex structure from a functional morphological perspective (Enomoto et al., 2024; Funaro et al., 2022; Handsfield et al., 2017; Knaus and Blemker, 2021; Shim et al., 2018). Because *in vivo* measurement or manipulation of Achilles tendon twist is technically challenging, functional morphological studies have primarily relied on *in vitro* and *in silico* studies. These studies have shown that a moderate degree of twist increases Achilles tendon strength (Shim et al., 2018), whereas a low degree of twist is associated with high local strain under loading (Funaro et al., 2022). However, previous studies have focused primarily on the mechanical behavior within the Achilles tendon itself, while the influence of tendon twist on force transmission from muscle to bone remains largely unexplored. A comprehensive understanding of Achilles tendon mechanics requires elucidating how its twisted structure contributes to force transmission—the fundamental function of tendons—and, ultimately, to joint motion generation.

The present study aimed to examine how the presence and degree of Achilles tendon twist influence the forces transmitted from the triceps surae to the skeleton, using finite element models with and without twist. We hypothesized that a twisted Achilles tendon generates non-axial force components at the distal end under a uniaxial proximally directed load, and that the magnitudes of these non-axial components increase with increasing twist.

## 2 Materials and methods

### 2.1 Model geometries

We created the three-dimensional free Achilles tendon geometry based on a previous study (Knaus and Blemker, 2021; Pękala et al., 2017) using the computer-aided design (CAD) software Fusion 360 (Autodesk Inc., San Francisco, CA, United States). How the individual subtendons insert onto the calcaneus—and how these insertion patterns vary with the degree of twist—has not been well documented. Therefore, in this study, we only modeled the Achilles tendon down to the superior border of its calcaneal insertion, the cross-sectional anatomy of which has been described in detail. The free tendon outline was generated by specifying elliptical contours at both ends of the model. The proximal ellipse measured 17.83 mm by 5.76 mm, and the distal ellipse measured 22.04 mm by 6.42 mm. These two contours were positioned 60 mm apart along the tendon’s long axis and were connected using a smoothly interpolated surface in the CAD environment to form the outer geometry. The proximal ellipse corresponded to the musculotendinous junction between the soleus muscle and the tendon, whereas distal ellipse represented the superior border of the insertion into the calcaneal bone of the Achilles tendon.

**FIGURE 1:**
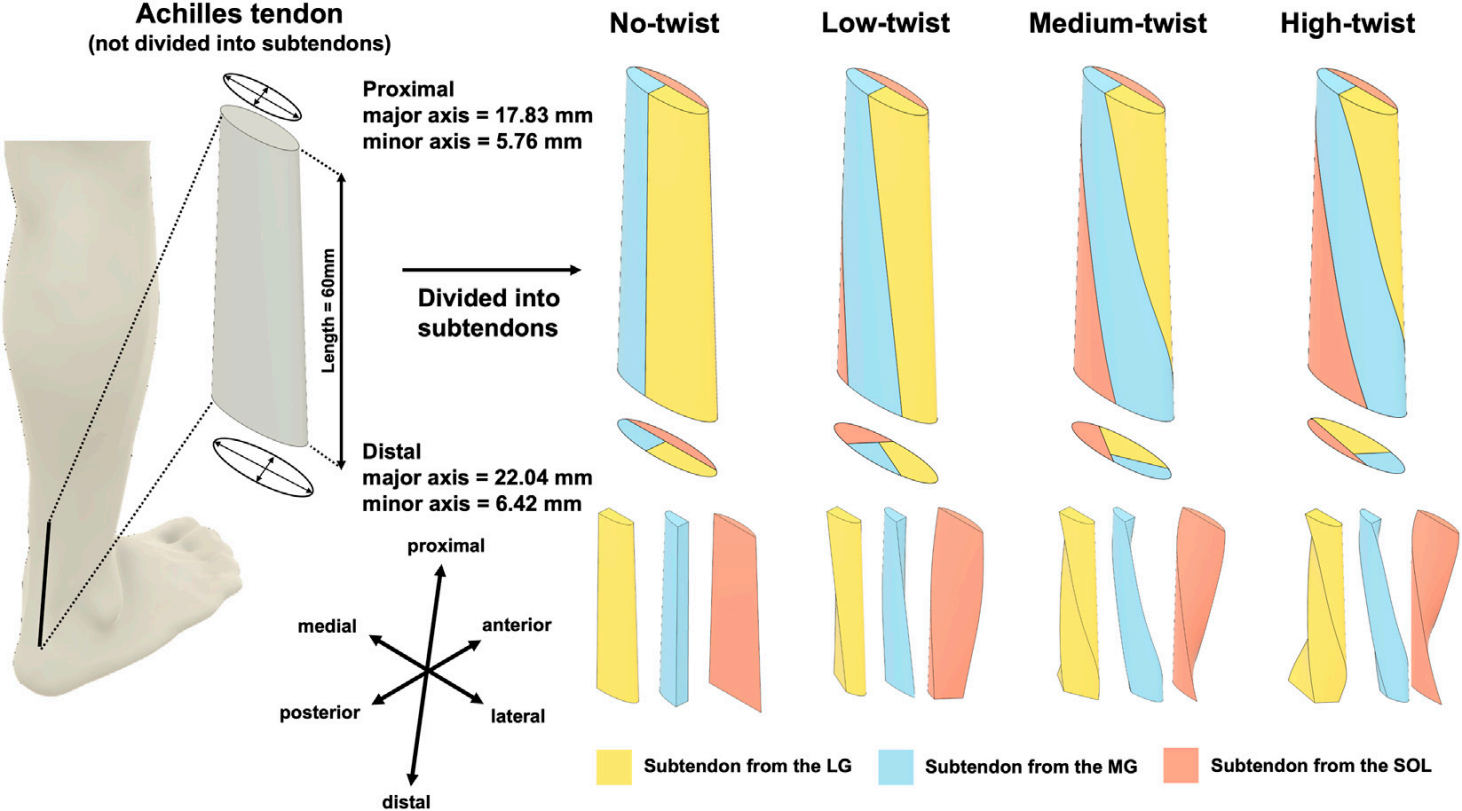
Achilles tendon models with four different degrees of twist. The Achilles tendon geometry prior to division into subtendons is shown in the upper left. The color-rendered Achilles tendon models on the right are divided into three subtendons, and the geometry of each subtendon is presented below each model. The foot image was generated using BodyParts3D/Anatomography (Database Center for Life Science, Japan) under the CC BY-SA 2.1 JP license. LG, lateral head of the gastrocnemius muscle; MG, medial head of the gastrocnemius muscle; SOL, soleus muscle.

Based on the Achilles tendon twist classification (Types 1–3) described by Pękala et al. (2017), the CAD model was divided into three subtendons: LG, MG, and SOL. Subtendon boundaries were established by embedding two curved internal surfaces that subdivided the tendon into three anatomical regions. Differences in twist were introduced by rotating the internal surfaces by prescribed angles. Reference lines indicating the intended subtendon boundaries were drawn on both the proximal and distal cross-sections. Each internal surface was generated by interpolating between paired reference lines located on the proximal and distal contours. In all models, the proximal shapes of the three subtendons were identical, while their distal geometries differed depending on the assigned twist pattern (Knaus and Blemker, 2021). The resulting twist magnitudes corresponded to three categories—low, moderate, and high—reflecting the average twist patterns described by Pękala et al. (2017). In addition, we created an Achilles tendon model without twist as a baseline. In total, we thus had four models with different degrees of twist (Figure 1). The fiber directions of each subtendon (oriented from the proximal to distal surfaces) were defined in FEBio Studio (Maas et al., 2012). The procedure for generating these geometries was similar to that used in previous studies (Enomoto et al., 2024; Knaus and Blemker, 2021).

### 2.2 Material properties

The Achilles tendon in this study was represented using a nearly incompressible transversely isotropic hyperelastic material (Weiss et al., 1996). The constitutive formulation implemented in FEBio (Maas et al., 2012) was adopted. The uncoupled strain energy function is expressed in Equation 1.

$$\Psi =F_{1}\left({\tilde{I}}_{1},{\tilde{I}}_{2}\right)+F_{2}\left(\tilde{\lambda }\right)+\frac{K}{2}{\left[\ln\left(J\right)\right]}^{2}$$ (1)

where ${\tilde{I}}_{1}$ and ${\tilde{I}}_{2}$ denote the first and second invariants, respectively, of the deviatoric variant of the right Cauchy-Green deformation tensor, while $\tilde{\lambda }$ represents the deviatoric part of the stretch along the fiber direction. The term *J =* det (F) is the Jacobian of the deformation. $F_{1}$ characterizes the material response of the isotropic ground substance matrix. $F_{2}$ represents the contribution from the fiber family. $F_{1}$ is taken to be a neo-Hookean energy and is given by $\frac{C_{1}\left(I_{1}-3\right)}{2}$ . The fiber stress is described in Equation 2.

$$\tilde{\lambda }\frac{\partial F_{2}}{\partial \tilde{\lambda }}=\left\{\begin{array}{c} 0\\ C_{3}\left(e^{C_{4}\left(\tilde{\lambda }-1\right)}-1\right)\\ C_{5}\tilde{\lambda }+C_{6} \end{array}\right.\begin{array}{c} \;\tilde{\lambda }\leq 1\\ \;1<\tilde{\lambda }<\lambda_{m}\\ \;\tilde{\lambda }\geq \lambda_{m} \end{array}$$ (2)

where $\lambda_{m}$ denotes the stretch level at which the fibers are straightened; The coefficients $C_{3}$ and $C_{4}$ are the scaling of the exponential stresses and rate of uncrimping of the fibers, respectively; $C_{5}$ corresponds to the modulus of straightened fibers. The constant $C_{6}$ is selected to ensure continuity of the stress at $\lambda_{m}$ . Material constants were obtained from a previous study (Shim et al., 2014) as follows: $C_{1}$ = 46.52 MPa, $C_{2}$ = 0, $C_{3}$ = 15.29 MPa, $C_{4}$ = 26.80, $C_{5}$ = 928.15 MPa. The value of $\lambda_{m}$ was set to 1.055 (Quapp and Weiss, 1998). The bulk modulus was assigned a value of 5.0 × 10^3^ MPa.

### 2.3 Boundary conditions

To simulate the load distribution arising from the triceps surae, we referred to the force-sharing ratios among the three muscles reported in previous studies (Crouzier et al., 2018; 2020). Based on these studies, the proportion of the force assigned to the LG, MG, and SOL subtendons was set to 10%, 25%, and 65%, respectively (Supplementary Table S1). A contractile force of 2000 N—an approximate value of the peak Achilles tendon force during walking (Kharazi et al., 2021)—was distributed to each subtendon according to these force-sharing ratios. The proximal surfaces of the subtendons were displaced only in the proximal–distal direction (z-axis). All degrees of freedom at the distal end were fixed to prevent motion.

Each Achilles subtendon is part of the larger Achilles tendon superstructure and does not have fully independent motion or function (Handsfield et al., 2020). In this study, based on the anatomical structure of the Achilles tendon and previous finite element studies (Diniz et al., 2023; Enomoto et al., 2024), the contact faces between the subtendons were not allowed to separate. Furthermore, *in vivo* experimental studies have shown that the Achilles subtendons can slide relative to each other during muscle contractions (Franz and Thelen, 2015; Slane and Thelen, 2015; Strand et al., 2025). However, the precise mechanical properties of the interfaces between subtendons remain unclear. Based on available experimental evidence and previous finite element studies (Diniz et al., 2023; Enomoto et al., 2024; Funaro et al., 2022; Knaus and Blemker, 2021), the contact faces between the three subtendons were modeled as frictionless to allow relative sliding between the subtendons. We performed all simulations quasistatically using FEBio Studio (Maas et al., 2012).

### 2.4 Mesh convergence test and validity of the model

Our model was meshed with tetrahedral second-order elements. A mesh convergence test was conducted by performing repeated simulations on low-twist models with varying mesh sizes. The mesh was considered sufficiently refined when further reductions in mesh size resulted in changes of less than 1% in the maximum elongation and sum of anterior–proximal force and medial–lateral force. We used an element size of 1 mm in our meshing process for every model.

Using the converged models, we tested the validity of the twisted models by comparing the shapes of the stress-strain curves and the maximum strains, as per experimental results reported in previous studies (Csapo et al., 2010; Kongsgaard et al., 2011).

### 2.5 Translational force and lateral force transmission

To examine how the presence and degree of twist affect the force transmitted to the distal end of the Achilles tendon, the resultant forces in the x-, y-, and z-directions were calculated at the fully constrained distal surface. The x-, y-, and z-components correspond to the medial–lateral, anterior–posterior, and proximal–distal directions, respectively. To evaluate lateral force transmission between subtendons, the proximal–distal force component was calculated for each subtendon. The difference between the proximally applied load and the distal reaction force (distal force minus proximal load) was then computed for each subtendon.

## 3 Results

### 3.1 Model validation

A previous experimental study reported that the peak Achilles tendon force during maximal voluntary isometric contraction of the plantar flexors reached 2,011 N, with an Achilles tendon strain of 0.045 ± 0.014 (Kongsgaard et al., 2011). The maximum strains observed in the low-, medium-, and high-twist models in this study were 0.0486, 0.0541, and 0.0599 when loaded with 2000 N, respectively, which were within approximately one standard deviation of the results reported in previous studies (Supplementary Figure S1). Our models exhibited nonlinear stress–strain curves, as reported in previous experimental studies (Csapo et al., 2010; Kongsgaard et al., 2011).

### 3.2 Three-dimensional force

**FIGURE 2:**
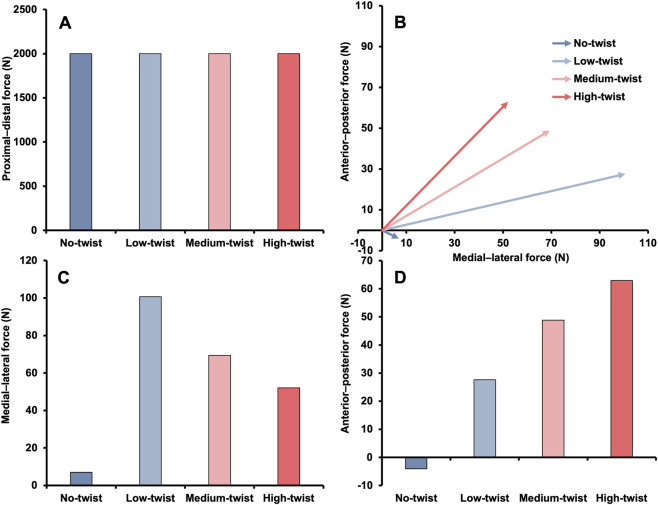
Forces observed at the distal end of the Achilles tendon. **(A)** Proximal–distal forces generated by each model. **(B)** Horizontal-plane force vectors calculated from each model. **(C)** Medial–lateral forces, and **(D)** anterior–posterior forces generated by each model. In the proximal–distal, anterior–posterior, and medial–lateral directions, positive values indicate proximal, anterior, and lateral components, respectively.

**FIGURE 3:**
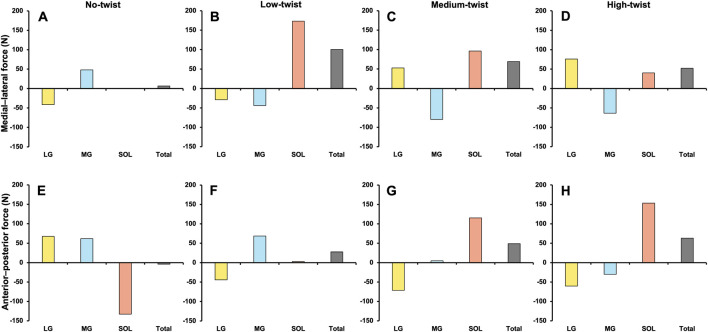
Forces observed at the distal end of each subtendon. **(A–D)** The upper panels show medial–lateral forces from each model, with positive values indicating lateral components. **(E–H)** The lower panels present anterior–posterior forces obtained from each model, with positive values indicating anterior components. Each panel shows the forces of the individual subtendons as well as their resultant force for the whole Achilles tendon. LG, lateral head of the gastrocnemius muscle; MG, medial head of the gastrocnemius muscle; SOL, soleus muscle.

Because a total tensile load of 2000 N was applied proximally in all simulations, an equal proximally directed reaction force of 2000 N was always observed at the distal end (Figure 2A). Introducing twist substantially altered the horizontal force components (Figures 2B–D). Whereas the no-twist model produced a negligible horizontal force, all twisted models generated clear anterolaterally directed forces (Figure 2B). Moreover, increasing the degree of twist caused the horizontal force vector to rotate progressively toward the anterior direction. In the medial–lateral direction, all models generated laterally directed forces. The low-twist model produced the largest such response, with the lateral force reaching approximately 101 N (Figure 2C). In the twisted models, the magnitude of the lateral force progressively decreased as the degree of twist increased. In the anterior–posterior direction, all twisted models produced anteriorly directed forces, and the magnitudes of these forces increased progressively with greater twist. The high-twist model showed the largest anterior force, reaching approximately 63 N (Figure 2D). The three subtendons produced force vectors of different magnitudes and directions, which combined to yield a single resultant force at the distal end (Figure 3).

### 3.3 Lateral force transmission and contact pressure

**FIGURE 4:**
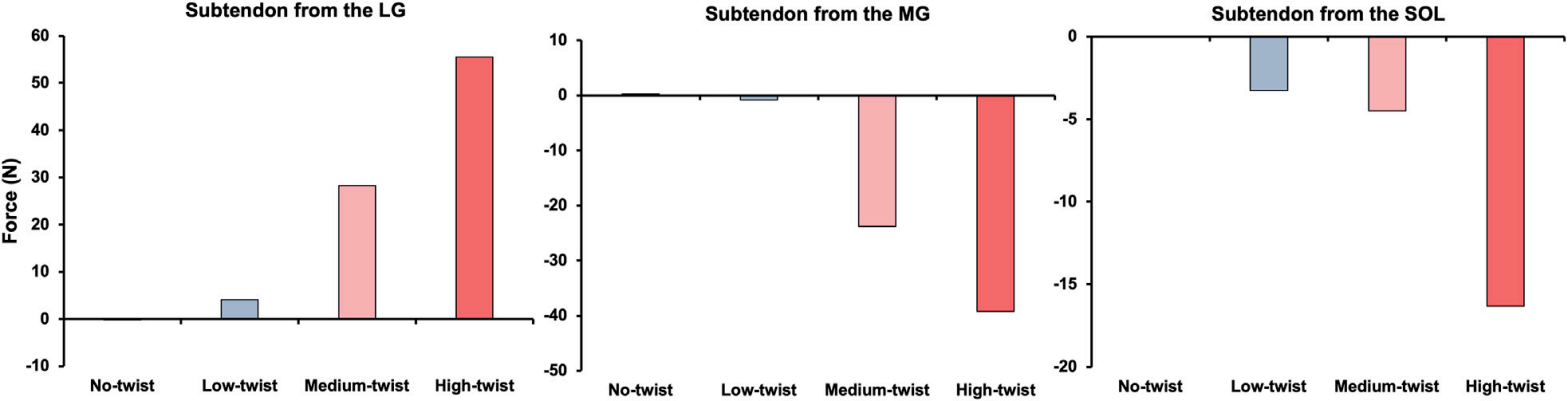
Difference in proximal–distal forces between the proximal and distal ends. Positive values indicate that the distal force is greater than the proximally applied load, and negative values indicate the opposite. LG, lateral head of the gastrocnemius muscle; MG, medial head of the gastrocnemius muscle; SOL, soleus muscle.

**FIGURE 5:**
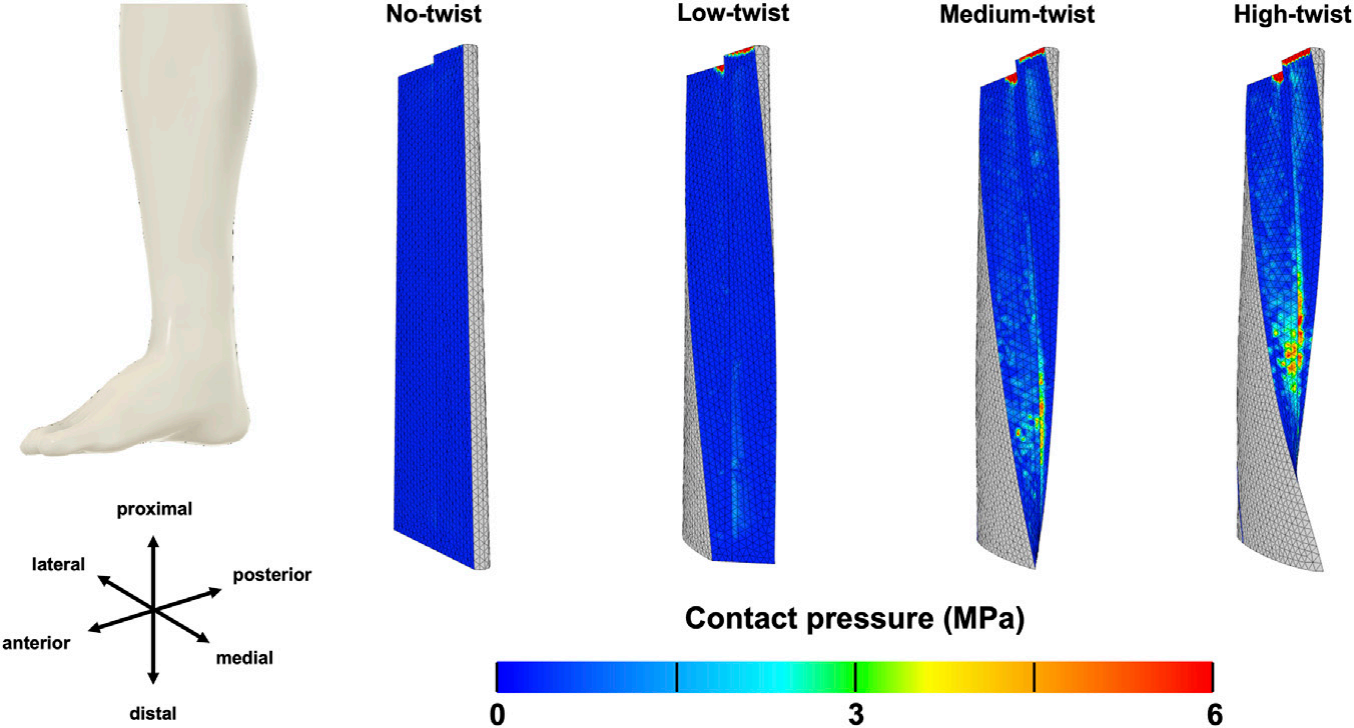
Contact pressure at the interfaces between subtendons. Each model is shown in an anterior–medial view, with the subtendon from the soleus hidden to visualize the contact surfaces. The foot image was generated using BodyParts3D/Anatomography (Database Center for Life Science, Japan) under the CC BY-SA 2.1 JP license.

Although the axial load and the distal reaction force were balanced in all models (Figure 2A), introducing twist induced axial force transmission between subtendons. In the no-twist model, the force applied at the proximal surface was transmitted to the distal surface almost unchanged (Figure 4). However, in the models with twist, the LG subtendon exhibited a larger distal force than the force applied proximally, whereas the MG and SOL subtendons showed the opposite pattern (Figure 4). The magnitudes of these differences increased progressively with greater twist. The contact pressure between subtendons, which is likely related to both the occurrence and magnitude of lateral force transmission, also increased progressively with greater twist (Figure 5).

## 4 Discussion

Our results show that the introduction of twist in the Achilles tendon substantially increases horizontal forces at its distal end. In addition, introducing twist promotes lateral force transmission between subtendons. In previous studies using finite element models (Cheung et al., 2005; Zhang et al., 2024) and musculoskeletal simulation software such as OpenSim (Delp et al., 2007), the force generated by the triceps surae was commonly modeled as a uniaxial force acting on the calcaneus. However, our results indicate that, in the presence of twist, the Achilles tendon transmits muscular forces in a complex three-dimensional manner through inter-subtendon interactions. Therefore, the force generated by the triceps surae may not be transmitted to the distal end of the Achilles tendon in a manner sufficiently simple for the above assumption to hold.

In contrast to the model without twist, the twisted models clearly exhibited anterolaterally directed horizontal forces at the distal end under a simple proximal tensile load. The lateral and anterior components reached approximately 101 N and 63 N, respectively. Moreover, not only the presence of twist but also its magnitude substantially affected the forces in the horizontal plane (Figure 2). These data indicate that the Achilles tendon with twist is not merely a tissue that transmits axially directed force but instead that the Achilles tendon pulls the calcaneus in an anterolateral direction. In generating horizontal forces, a particularly important feature is that the SOL subtendon, which bears the largest portion of the applied tensile load, shifts its distal position substantially depending on the degree of twist. Although the SOL subtendon is located anteriorly and near the midline in the mediolateral direction at the proximal surface, it shifts medially at the distal end in the low-twist model (Figure 1). This medial shift, in turn, pulls the distal end strongly in the lateral direction (Figure 3B). In contrast, as the degree of twist increases, the SOL subtendon progressively shifts posteriorly (Figure 1), leading to a decrease in laterally directed force (Figures 3B–D) and an increase in anteriorly directed force (Figures 3F–H). However, the SOL subtendon does not solely account for the generation of horizontal force. Rather, all three subtendons produce force components of different magnitudes and directions, ultimately resulting in a single net horizontal force at the distal end (Figure 3). Thus, horizontal force at the distal end arises from interactions between the loads borne by each subtendon and their relative positions. Through these inter-subtendon interactions, the Achilles tendon transmits force in a complex three-dimensional manner.

We studied how the horizontal forces observed in this study influence foot mechanics by focusing on vertical free moment (VFM). VFM is defined as the moment about a vertical axis acting at the center of pressure (COP) that arises from foot–ground friction, and it has been reported to contribute to the dynamic stability and efficiency of walking (Negishi and Ogihara, 2023). When we computed the VFM around the COP generated by the horizontally directed forces under simplified assumptions, the low-twist model produced a maximum torque of 17.1 Nm, and this value decreased progressively with increasing twist (Supplementary Figure S2). Previous studies have reported that the peak VFM during walking, normalized to body weight, is approximately 5.11 × 10^−3^ Nm/body weight in magnitude (Ohkawa et al., 2017). When normalized to a body weight of 700 N, our calculation yielded a value of 24.43 × 10^−3^ Nm/body weight, which exceeds the value reported in prior work. Because the VFM in this study was computed under simplified assumptions, the magnitudes should not be directly compared. In addition, because information on subtendon morphology at the calcaneal insertion is lacking, we only modeled the Achilles tendon down to the superior border of its insertion into the calcaneus. Therefore, whether the distal forces observed here are transmitted directly to the calcaneus should be interpreted with caution. Previous work has reported that the relative positions of individual subtendons do not shift substantially between the superior border of the calcaneal insertion and the actual insertion of the Achilles tendon (Pękala et al., 2017). Taken together, the horizontal forces generated by the twisted Achilles tendon are likely to have a nonnegligible influence on the generation of VFM. Numerous recent finite element studies have employed anatomically detailed foot models (Ito et al., 2021; 2026; Yanguma-Muñoz et al., 2024; Zhang et al., 2024). Future studies integrating the twisted Achilles tendon model with such foot finite element models should assess how these horizontal forces affect foot mechanics and whole-body dynamics.

The subtendons of the Achilles tendon transmit axial forces to one another under loading (Gains et al., 2020). This inter-subtendon force transmission has been reported to play a role in distributing loads across larger cross-sectional areas, thereby reducing peak stresses and diminishing the risk of tissue overload (Maas and Finni, 2018). Our results show that, even when the subtendon surfaces were taken to be frictionless, the twisted Achilles tendon still transmitted axial forces between subtendons, which in turn altered the magnitudes of forces observed at the distal ends (Figure 4). Furthermore, the magnitude of this alteration increased progressively with greater degrees of twist (Figure 4). The LG subtendon in the high-twist model showed the largest change, exhibiting a 27.78% increase in axial force: when 200 N was applied proximally, a force of 255.55 N was observed at the distal end. To date, lateral force transmission within tendons has been discussed primarily in the context of force transfer properties of the interfascicular matrix (Haraldsson et al., 2008; Thorpe et al., 2015) or the inter-subtendon matrix (Gains et al., 2020). For example, Gains et al. (2020) reported that the presence of an inter-subtendon matrix enables adjacent subtendons to transmit axial forces *in vitro*. In contrast, our model demonstrated force transmission between subtendons even when the inter-subtendon surfaces were frictionless—that is, when the presence of an inter-subtendon matrix was omitted. These findings suggest that force transmission between subtendons may arise not only from the inter-subtendon matrix but also from morphological features of the twist. We also found that introducing twist generated contact pressure at the subtendon interfaces and that this pressure increased progressively with greater degrees of twist (Figure 5). This pattern is consistent with a previous experimental study reporting that intratendinous pressure of the Achilles tendon increases under loading as the degree of twist increases (Pringels et al., 2023). These results indicate that twist induces interfacial compression between subtendons, effectively pressing their contacting surfaces more firmly together, and that this tightening effect intensifies with greater twist. In addition, lateral force transmission is likely facilitated by the unbalanced contractile forces generated by the triceps surae: the more heavily loaded MG and SOL subtendons drive the lightly loaded LG subtendon upward, thereby contributing to horizontal force transfer.

In the following section, we highlight the key assumptions in our modeling approach and outline several issues that warrant further investigation. We modeled an Achilles tendon using a simple, idealized geometry. Previous finite element studies using ultrasound-based subject-specific models have shown that the Achilles tendon geometry can affect its mechanical behavior (Shim et al., 2014). Therefore, the simplified geometry used in the present study may have influenced the main outcomes of the present study, including horizontal forces, inter-subtendon force transmission, and contact pressure. These results should be interpreted with caution accordingly. Moreover, previous studies have reported substantial inter-individual variation in Achilles tendon morphology, including in length, thickness, and width, and these geometric differences affect mechanical behavior within the tendon (Enomoto and Oda, 2023; Enomoto et al., 2024). Moreover, recent work has increasingly focused on the extent to which subtendons can slide relative to one another (Franz and Thelen, 2015; Slane and Thelen, 2015; Strand et al., 2025). Previous *in vivo* ultrasound studies have reported that the extent of inter-subtendon sliding is influenced by aging (Franz and Thelen, 2015) and by sustaining an injury (Couppé et al., 2020; Lecompte et al., 2024b). In addition, recent finite element analysis studies have shown that both the presence and magnitude of friction between subtendons alter mechanical behavior within the Achilles tendon (Handsfield et al., 2017; Yin et al., 2021). Thus, future work should investigate how morphological and mechanical variations of the Achilles tendon—beyond twist alone—affect its force transmission properties using more anatomically detailed models. We assessed the validity of our finite element model using the maximum tendon strains and stress-strain curves. However, because of a lack of experimental data, we were unable to validate the predicted horizontal forces, inter-subtendon force transmission, and contact pressure. Future validation using *in vivo* data will be needed to support the interpretation of these model-based predictions. Although our results suggest that a twisted Achilles tendon can generate forces capable of displacing the calcaneus anterolaterally, even the maximum magnitude of this force was only approximately 5% of the axial force. Furthermore, we were not able to accurately assess how these horizontally directed forces influence foot dynamics or mechanical interactions with the external environment. Finally, in this study we modeled the loading applied to the Achilles tendon during walking. During running and jumping, however, the tendon experiences substantially stronger forces under highly dynamic conditions (Fukashiro et al., 1995; Komi, 1990; Reiter et al., 2024). Investigating tendon response under such high-load, dynamic conditions will provide a more detailed understanding of its functional role.

## 5 Conclusion

Our results suggest that the uniaxial contractile force generated by the triceps surae is transformed into triaxial forces by the twisted Achilles tendon and is redistributed in a complex three-dimensional manner through inter-subtendon interactions. These findings provide foundational insight into the force transmission properties of the Achilles tendon. Recently, high-field magnetic resonance imaging and ultrasound imaging have increasingly been used to quantify the degree of twist and to characterize subtendon morphology *in vivo* (Cone et al., 2023; Finni et al., 2025; Lecompte et al., 2024a). This line of research is likely to continue developing as efforts expand to examine how the degree of Achilles tendon twist relates to sports performance and injury risk. When interpreting such findings, an understanding of how twist influences the tendon’s fundamental ability to transmit force may be important. Although this study has several limitations, including model simplifications and limited experimental validation, it provides a mechanistic basis for understanding the mechanical functions of the twisted morphology of the Achilles tendon. Future work should investigate how the forces transmitted through the twisted tendon, arising from the contractile output of the triceps surae, affect the dynamics of the foot and the body as a whole.

## Data Availability

The original contributions presented in the study are included in the article/Supplementary Material, further inquiries can be directed to the corresponding author.
