# Supplementary material for: High horizontal force and lateral force transmission induced by the twist of the Achilles tendon
Source: Front Bioeng Biotechnol. 2026 Jul 23;14:1874501. doi: 10.3389/fbioe.2026.1874501 (PMC13442520; doi:10.3389/fbioe.2026.1874501)
Supplement: Supplementary file 1 [file DataSheet1.docx]

Supplementary Material

# 1 Supplementary Figures and Tables

## 1.1 Supplementary Figures

**Supplementary Figure S1** Stress–strain curves of the low-, medium-, and high-twist models. The vertical line in the middle indicates the peak Achilles tendon strain during maximal voluntary isometric contraction of the plantar flexors reported in a previous study (Kongsgaard et al., 2011). Additional vertical lines on either side indicate plus and minus one standard deviation of the peak Achilles tendon strain reported in the previous study (Kongsgaard et al., 2011).

**Supplementary Figure S2** Vertical free moment (VFM) around the center of pressure (COP) generated by the horizontal forces in each model. In this study, the peak Achilles tendon force during walking (Kharazi et al., 2021) was applied to all models. During the push-off phase of walking, when peak Achilles tendon force is thought to occur, the COP has been reported to be located approximately 0.17 m anterior to the posterior end of the foot and near the midpoint in the medial–lateral direction (Li et al., 2020). Based on this assumption, panel **A** presents a schematic diagram illustrating the spatial relationship between the COP and the horizontal forces generated by each model when applied to the right heel. This panel shows a top view of the right foot. Using these parameters and the following equation, the VFM around the COP was calculated (**B**).

$$VFM= moment arm\times f_{lateral}$$

where $moment arm$ and $f_{lateral}$ denote the anterior–posterior distance from the posterior end of the calcaneus to the COP (0.17 m) and the lateral force generated in each model, respectively. As mentioned above, because the COP is located near the midpoint in the medial–lateral direction during the push-off phase of walking (Li et al., 2020), the moment arm in the medial–lateral direction and the anterior–posterior force were ignored. It should also be noted that in this calculation it was assumed that the horizontal forces obtained at the distal end of the Achilles tendon model are transmitted directly to the posterior end of the calcaneus, and that changes in the moment arm due to calcaneal lift-off during the latter half of the stance phase were ignored. The foot image was generated using BodyParts3D/Anatomography (Database Center for Life Science, Japan) under the CC BY-SA 2.1 JP license. COP, center of pressure.

**Supplementary Table S1** The force-sharing ratio of the triceps surae reported in previous studies and used in this study.

| Study | LG/TS (%) | MG/TS (%) | SOL/TS (%) |
| --- | --- | --- | --- |
| Crouzier et al. (2020): 20%MVC | 7.5 | 22.7 | 69.8 |
| Crouzier et al. (2020): 40%MVC | 12.1 | 25.9 | 62 |
| Crouzier et al. (2018): 20%MVC | 6 | 26.2 | 67.8 |
| This study | 10 | 25 | 65 |

Each value indicates the force ratio of each head of the triceps surae during muscle contraction. The ratio used in this study was determined based on the three values reported in previous studies. TS, triceps surae; LG, lateral head of the gastrocnemius muscle; MG, medial head of the gastrocnemius muscle; SOL, soleus muscle; MVC, maximum voluntary contraction

References

Crouzier, M., Lacourpaille, L., Nordez, A., Tucker, K., and Hug, F. (2018). Neuromechanical coupling within the human triceps surae and its consequence on individual force-sharing strategies. *J. Exp. Biol.* 221, jeb187260. doi: 10.1242/jeb.187260

Crouzier, M., Tucker, K., Lacourpaille, L., Doguet, V., Fayet, G., Dauty, M., et al. (2020). Force-sharing within the triceps surae: an Achilles heel in Achilles tendinopathy. *Med. Sci. Sports Exerc.* 52, 1076–1087. doi: 10.1249/MSS.0000000000002229

Kharazi, M., Bohm, S., Theodorakis, C., Mersmann, F., and Arampatzis, A. (2021). Quantifying mechanical loading and elastic strain energy of the human Achilles tendon during walking and running. *Sci. Rep.* 11, 5830. doi: 10.1038/s41598-021-84847-w

Kongsgaard, M., Nielsen, C. H., Hegnsvad, S., Aagaard, P., and Magnusson, S. P. (2011). Mechanical properties of the human Achilles tendon, in vivo. *Clin. Biomech.* 26, 772–777. doi: 10.1016/j.clinbiomech.2011.02.011

Li, B., Xiang, Q., and Zhang, X. (2020). The center of pressure progression characterizes the dynamic function of high-arched feet during walking. *J. Leather Sci. Eng.* 2, 1. doi: 10.1186/s42825-019-0016-6
